# Recommendations for Digital Single‐Operator Cholangiopancreatoscopy: Turning Vision Into Practice!

**DOI:** 10.1002/ueg2.70109

**Published:** 2025-09-08

**Authors:** Michiel Bronswijk, Giuseppe Vanella

**Affiliations:** ^1^ Department of Gastroenterology and Hepatology Imelda General Hospital Bonheiden Belgium; ^2^ Imelda Clinical GI Research Center Bonheiden Belgium; ^3^ IRCCS San Raffaele Scientific Institute and Vita‐Salute University Milan Italy

**Abbreviations**
dSOC: digital single‐operator cholangioscopy
dSOP: digital single‐operator pancreatoscopy
ERCP: endoscopic retrograde cholangiopancreatography
MD‐IPMN: main‐duct intraductal papillary mucinous neoplasm
NSAID: non‐steroidal anti‐inflammatory drugs

Navigation, diagnosis and therapeutics during ERCP are often hampered by fluoroscopy‐based 2D visualization. Cholangiopancreatoscopy has facilitated a major leap forward, by enabling direct optical visualization, guiding a wide range of diagnostic and therapeutic implementations. Further technical developments beyond the historical “mother‐baby cholangioscopy” have widely led to an incorporation of cholangioscopy in treatment algorithms [1, 2]. However, as always with new technology, limited guidance is available on when and how to implement digital single‐operator cholangioscopy (d‐SOC) or pancreatoscopy (d‐SOP) [3, 4, 5].

We therefore applaud the efforts of the European Cholangioscopy Group who, in this issue [6] of *UEG Journal,* present their consensus recommendations on general principles, as well as implementation in the management of pancreatobiliary stones, indeterminate strictures and other indications. Under the leadership of authors David De Jong and Pieter Hyndrickx, the Delphi process was completed by 30 out of 32 cholangioscopy experts using an online survey. Employing anonymized feedback and an 80% approval threshold, 39 out of 43 statements were accepted in the first Delphi round, followed by approval of 1 and 2 statements in subsequent rounds respectively (mean acceptance rate 93.3%).

The positioning of these 42 recommendations within available guidance warrants further consideration. Compared to available guidelines, the present consensus document may offer a substantially more topic‐specific guidance on various cholangiopancreatoscopy areas. (1) Peri‐procedural considerations: Antimicrobial and NSAID prophylaxis, as well as on scope introduction and manipulation, are described in depth and more specific guidance is provided on these topics [3, 4, 7]. Crucial points for colleagues in the beginning of the learning curve as well as established endoscopists. (2) Alternative access strategies: The consensus furthermore provides innovative statements on alternatives to retrograde access, suggesting that percutaneous cholangioscopy and antegrade pancreatoscopy can be considered should ERCP fail. This adequately reflects the current approach in many referral centers throughout Europe, selecting alternative access techniques early on, when clinically appropriate [8, 9]. However, these techniques require a high level of technical skill and should be employed in high volume setting following careful consideration. For the time being, this seems to be the first consensus document to explicitly endorse both percutaneous cholangioscopy and antegrade pancreatoscopy in favorable situations [7], hereby expanding the guidance on complex hepatopancreatobiliary disease management. (3) Pancreatic diagnosis: The panel of experts suggests that d‐SOP can be considered in the evaluation of main duct dilation or for the perioperative assessment of main‐duct intraductal papillary mucinous neoplasms (MD‐IPMN). (4) Initial work‐up unexplained biliary strictures: the authors furthermore suggest that d‐SOC should be considered in the initial work‐up of unexplained biliary strictures if expertise is available, hereby potentially reducing the need for additional diagnostic procedures and optimizing the diagnostic process. When compared to recently published guidelines [1, 2], the current consensus therefore seems to allow for a more prominent role for cholangioscopy at the index procedure.

Beyond the abovementioned indications, cholangiopancreatoscopy has proven its worth in various auxiliary roles, such as hemobilia, wire cannulation and extraction of foreign bodies or migrated stents. Applications also acknowledged throughout the current recommendations.

Limitations of this consensus document mainly relate to the potential geographical preferences and practice patterns, as alternative pathways or techniques might be favored in different regions worldwide. Accessibility and cost continue to present important considerations in cholangiopancreatoscopy, and the effect of evolving market forces on device cost are likely to become apparent in the coming years. We furthermore take note of a very high first‐round acceptance rate, which might raise questions on the influence of online voting versus in‐person consensus meetings. Nevertheless, the authors also assessed this particular aspect, providing detailed information on first round feedback as well as subsequent rounds.

We can safely say that this consensus document has addressed an extensive array of topics and provides an up‐to‐date guidance within cholangiopancreatoscopy, including aspects that larger, established societies historically might not have covered. Nonetheless, it is also quite clear that more studies on various cholangiopancreatoscopy topics are warranted, such as improvement of optical diagnosis, with or without artificial intelligence [10], implementation in pancreatic stone and stricture management [11], as well as complex stone management [12]. Reflecting at the coming of age of cholangiopancreatoscopy, its trajectory foreshadows an era in which direct optical assessment will be integral to pancreatobiliary endoscopy, instead of purely ancillary. An evolution further strengthened by recommendations such as the current one.

## Conflicts of Interest

The authors declare no conflicts of interest.

## Data Availability

The authors have nothing to report.
